# Resistive switching memory performance in oxide hetero-nanocrystals with well-controlled interfaces

**DOI:** 10.1080/14686996.2020.1736948

**Published:** 2020-03-19

**Authors:** Takafumi Ishibe, Yoshiki Maeda, Tsukasa Terada, Nobuyasu Naruse, Yutaka Mera, Eiichi Kobayashi, Yoshiaki Nakamura

**Affiliations:** a Graduate School of Engineering Science, Osaka University, Toyonaka, Osaka, Japan; b Department of Fundamental Bioscience, Shiga University of Medical Science, Otsu, Shiga, Japan; c Kyushu Synchrotron Light Research Center, Tosu, Saga, Japan

**Keywords:** Memristor, interface control, nanocrystal, iron oxide, silicon, germanium, resistive switching characteristics, 212 Surface and interfaces

## Abstract

For realization of new informative systems, the memristor working like synapse has drawn much attention. We developed isolated high-density Fe_3_O_4_ nanocrystals on Ge nuclei/Si with uniform and high resistive switching performance using low-temperature growth. The Fe_3_O_4_ nanocrystals on Ge nuclei had a well-controlled interface (Fe_3_O_4_/GeO_x_/Ge) composed of high-crystallinity Fe_3_O_4_ and high-quality GeO_x_ layers. The nanocrystals showed uniform resistive switching characteristics (high switching probability of ~90%) and relatively high Off/On resistance ratio (~58). The high-quality interface enables electric field application to Fe_3_O_4_ and GeO_x_ near the interface, which leads to effective positively charged oxygen vacancy movement, resulting in high-performance resistive switching. Furthermore, we successfully observed memory effect in nanocrystals with well-controlled interface. The experimental confirmation of the memory effect existence even in ultrasmall nanocrystals is significant for realizing non-volatile nanocrystal memory leading to neuromorphic devices.

## Graphical abstract

**Figure:**
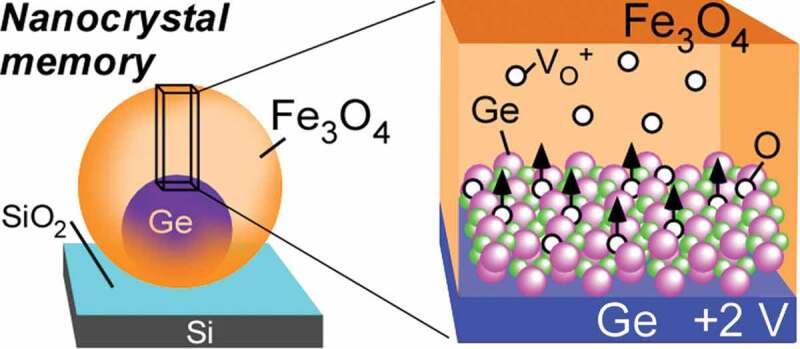

## 1. Introduction

New informative systems toward next-generation human society such as artificial intelligence and internet of things have been extensively studied. Now, the systems for huge, fast, and complex calculations are requiring neuromorphic devices [1]. A lot of researchers have enthusiastically paid attention to memristor working like synapse: i.e. memorizing an electric current history [2,3]. Resistance random access memory (ReRAM), which can realize memristor, has great advantages such as high-speed response, low power consumption, and good scalability [4–10].

The resistive switching characteristics were mainly observed in oxide-based materials such as TaO_x_ [11], TiO_x_ [12,13], and so on [14–16]. Fe_3_O_4_ with a higher Clark number (Fe is rank 4^th^) is a more industrially compatible material in terms of element abundance, low material cost, and non-toxicity [17,18]. Polycrystalline Fe_3_O_4_ film showed resistive switching characteristics based on ionic movement [19,20]. However, the Off/On resistance ratio (Off/On ratio) is low (~10) because the leakage current passing through the grain boundaries may degrade the Off resistance. Therefore, forming high-quality Fe_3_O_4_ is a key for increasing Off/On ratio.

**Figure 1.**
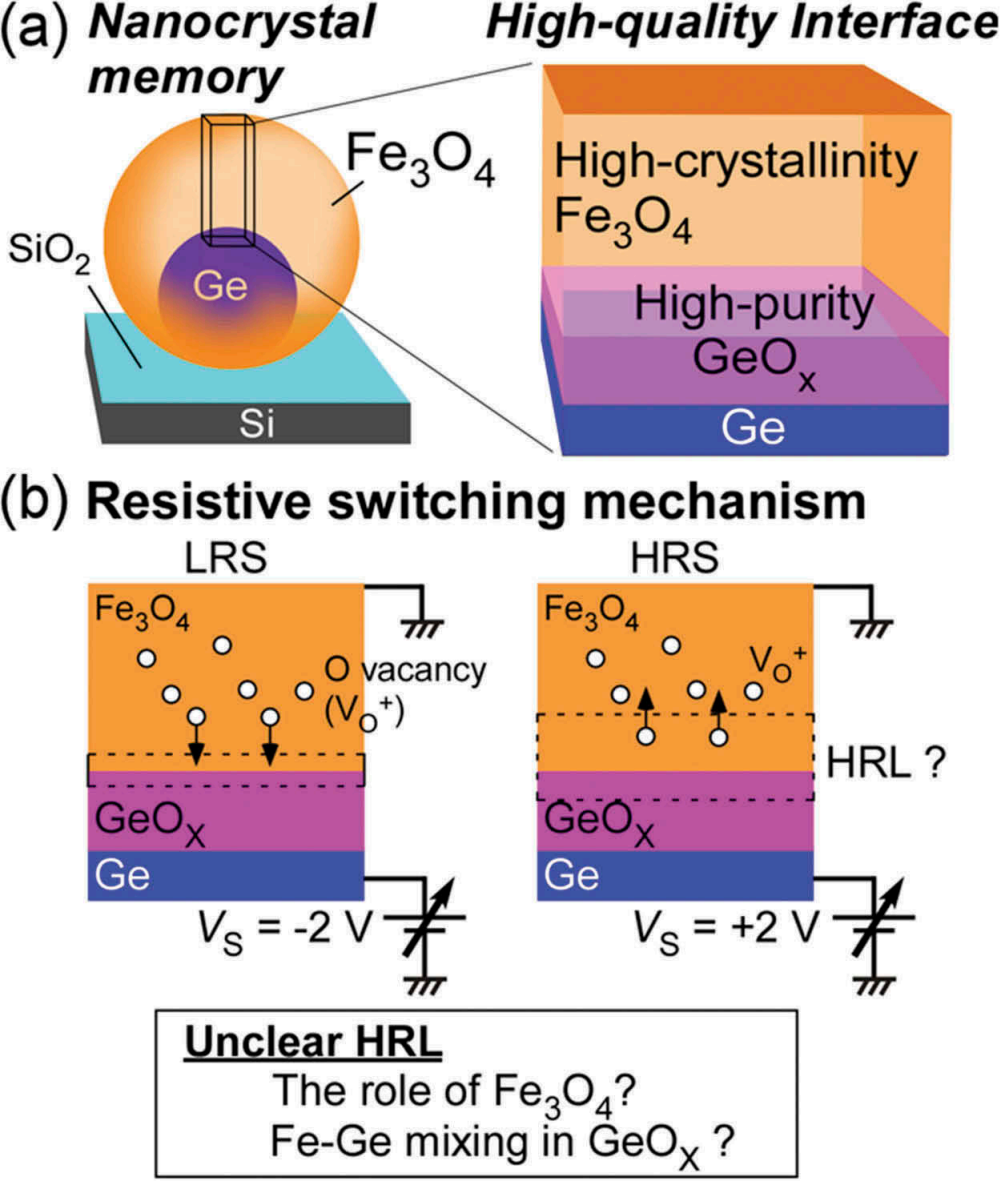
(a) Schematic diagram of isolated Fe_3_O_4_/GeO_x_/Ge NC memory (left image). The formation of high-crystallinity Fe_3_O_4_ and high-purity GeO_x_ at the interface between Fe_3_O_4_ and Ge (right image) aiming at high resistive switching performance. (b) Resistive switching mechanism in Fe_3_O_4_/GeO_x_/Ge NCs that have some unclearness: high resistance layer (HRL) which is a key for nanoionics-based resistive switching

Nanostructuring approach can be one of the ways to form high-crystallinity materials. We have developed single-crystalline spherical nanocrystals (NCs), where there are almost no defects due to the elastic relaxation of the lattice mismatch strain between NCs and substrates [21]. Hence, the NCs without current leak paths likely show higher Off resistance, leading to higher Off/On ratio. Recently, we have developed core/shell epitaxial Fe_3_O_4_/Ge NCs by combining oblique deposition at high temperature with ultrathin SiO_2_ technique [22–25] (left image in Figure 1(a)), where Ge oxides (GeO_x_) were formed at the Fe_3_O_4_/Ge interface by exposure to oxygen atmosphere after epitaxial growth of Fe_3_O_4_ (at the final stage of Fe_3_O_4_ growth or when exposing to air). Its detail is described in our previous study [17]. The Fe_3_O_4_/Ge NCs exhibited high Off/On ratio of ~100 [26], but had large positional variability. To control the resistive switching characteristics well, understanding the mechanism is strongly required. So far, it has been revealed that the resistive switching is originated from the thickness change of high resistance layer (HRL) near Fe_3_O_4_/GeO_x_/Ge interface caused by electric-field-induced oxygen vacancy movement (Figure 1(b)). However, the identity of HRL, a key of this nanoionics-based resistive switching, has not been clarified: in the interface layer, existence or non-existence of Fe-Ge mixing, and oxidation degree, etc. The mechanism unclearness related to poor resistive switching performance is ascribed to the unclear and uncontrolled Fe_3_O_4_/GeO_x_/Ge interface.

In this study, aiming at forming well-controlled Fe_3_O_4_/GeO_x_/Ge interface in the NC system, we perform low-temperature growth of epitaxial Fe_3_O_4_ NCs without the interface reaction degrading the performance by depositing Fe on Ge nuclei at room temperature (RT) under a low-pressure oxygen atmosphere and subsequently post-annealing. Then, we obtain Fe_3_O_4_/GeO_x_/Ge NCs, where Fe_3_O_4_ is highly crystalline and the high-quality GeO_x_ is formed between Fe_3_O_4_ and Ge. The post-annealed NCs showed uniform resistive switching characteristics (higher switching probability of ~90%) and higher Off/On ratio of ~58. It is found that HRL related to resistive switching mechanism is composed of Fe_3_O_4_ near the interface and GeO. Furthermore, we successfully observe the memory effect in the NCs with well-controlled interface. The experimental confirmation of the memory effect existence even in ultrasmall NCs is significant for realizing non-volatile NC memory leading to neuromorphic device.

## 2. Experimental details

Fe_3_O_4_ NCs on Ge nuclei were formed on Si substrates in the chamber equipped with Knudsen cell for Ge and an electron beam evaporator for Fe in the following way. As-doped Si(111) substrates with dimensions of 2 mm×7 mm×0.3 mm were introduced into ultrahigh vacuum chamber at a base pressure of 1 × 10^−8^ Pa. After degassing the substrates at 500°C for 6 h, Si(111) clean surfaces were obtained by flashing at 1250°C. The ultrathin SiO_2_ films with the thickness of 0.3 nm were formed on Si(111) substrates by oxidizing the Si surfaces at 600°C for 10 min under an oxygen pressure of 2 × 10^−4^ Pa [17,21–26]. Epitaxial Ge nuclei with an areal density of ~10^11^ cm^−2^ were formed by depositing 25 MLs of Ge onto the ultrathin SiO_2_ films at 600°C. 1–30 MLs of Fe were deposited on Ge nuclei at RT for coating Ge nucleus surfaces. Finally, Fe_3_O_4_ NCs were grown on Fe-coated Ge nuclei by deposition of 21 ML Fe on the Ge nuclei at RT under an oxygen pressure of 2 × 10^−4^ Pa. In this Fe_3_O_4_ growth by Fe deposition at low-pressure oxygen atmosphere, oxygen vacancies are likely generated in Fe_3_O_4_ [27,28]. For the above-mentioned Fe coating and Fe_3_O_4_ growth, the oblique Fe deposition was performed in the direction of out-of-plane incident angle of 25º, enabling the formation of isolated Fe_3_O_4_ NCs on Ge nuclei, which details are reported in our previous study [26]. For Fe and Ge deposition, Fe and Ge fluxes are 0.13 and 0.35 ML/min, respectively. To enhance their crystallinities, Fe_3_O_4_ NCs were post-annealed at 250-400°C for 30 min under an oxygen pressure of 2 × 10^−4^ Pa.

Reflection high energy electron diffraction (RHEED) observations were performed with 13 keV electron beam incident in the <112> _Si_ direction. The structures of Fe_3_O_4_/GeO_x_/Ge NCs were observed by cross-sectional high-resolution transmission electron microscopy (HRTEM) with 200 keV electron beam incident in the direction of <10> _Si_. GeO_x_ layers were likely formed at the Fe_3_O_4_/Ge interface at the final stage of Fe_3_O_4_ growth or after exposure to oxygen atmosphere. To determine the compositions of the aforementioned interfaces, we performed XPS measurements in the Fe2p and Ge3d regions.

Conductive-atomic force microscopy (C-AFM) measurements were carried out using a Pt-Ir-coated Si cantilever at RT in air. For absolute current (*I*_S_)-sample bias voltage (*V*_S_) measurement, the *V*_S_ was swept at the rising and falling speed of 0.4 V/s and the voltage step of 0.006 V in the following order: (1) 0 V → 2 V, (2) 2 V → 0 V, (3) 0 V → −2 V, and (4) −2 V → 0 V.

## 3. Results and discussions

### 3.1. Resistive switching characteristics in the NCs grown at RT

**Figure 2.**
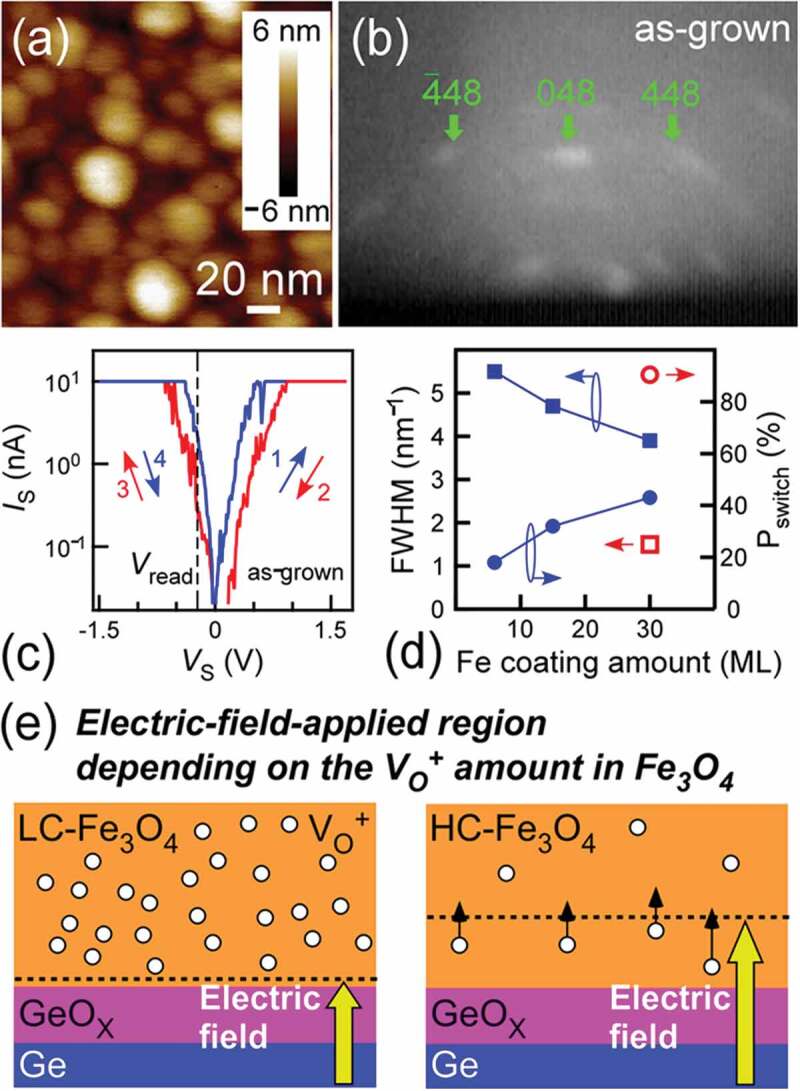
(a) AFM image, (b) RHEED pattern, and (c) The *I*_S_-*V*_S_ curve of as-grown NCs with the Fe coating layer of 30 MLs. The *V*_S_ was swept in the following order: (1) 0V→ 2V, (2) 2V → 0V, (3) 0V → −2V, and (4) −2V → 0V. (d) FWHM of 224_Fe3O4_ (left axis) and *P*_switch_ (right axis) as a function of the deposition amount of Fe coating layer in as-grown NCs (the solid squares and circles, respectively). The open square and circle denote the FWHM of 224_Fe3O4_ and *P*_switch_ in NCs annealed at 250°C, respectively. (e) Schematic of the Fe_3_O_4_ crystallinity effect on the resistive switching characteristics, where low- and high-crystallinity Fe_3_O_4_ are denoted as LC-Fe_3_O_4_ and HC-Fe_3_O_4_. Electric-field-applied region gets smaller in Fe_3_O_4_ with more oxygen vacancies (V_O_^+^) because of large screening effect by high concentration carriers

The morphologies of the NC samples grown at RT (as-grown NCs) with various Fe coating layers were measured by AFM. An AFM image of as-grown NCs with Fe coating layer of 30 MLs in Figure 2(a) indicates the formation of isolated NCs that have high areal density of ~10^11^ cm^−2^ and small size of ~20-40 nm. From RHEED observations, it was confirmed that Fe_3_O_4_ NCs were epitaxially grown on Ge nuclei/Si (Figure 2(b)), which detailed RHEED analyses are reported in our previous studies [17,26]. The analyses of 224_Fe3O4_ spots in RHEED patterns were performed by fitting with Gaussian function. The full width at half maximum (FWHM) values of the 224_Fe3O4_ fitted peaks in Fe_3_O_4_ NCs on Ge nuclei with Fe coating layers of 6, 15, and 30 MLs were 5.5, 4.7, and 3.9 nm^−1^, respectively (Figure 2(d) and Supplemental Material 1), indicating that the crystallinity is enhanced with increasing the deposition amount of Fe coating layer. This trend is similar to the previously reported result in Fe_3_O_4_ NCs on Ge nuclei formed by Fe deposition at high temperature [29], where it was reported that the oxidation of Ge nucleus surface at the first stage of Fe_3_O_4_ growth was prevented by Fe coating, resulting in the higher crystallinity of Fe_3_O_4_. Therein, at the final stage of Fe_3_O_4_ growth or after exposure to oxygen atmosphere, the GeO_x_ was inserted at the Fe_3_O_4_/Ge interface [30]. Therefore, it is considered that as-grown NCs also have GeO_x_ at the Fe_3_O_4_/Ge interface.

The resistive switching characteristics in as-grown NCs with Fe coating layers of 6, 15, and 30 MLs were measured using C-AFM. The *V*_S_ was swept in the following order: (1) 0 V → 2 V, (2) 2 V → 0 V, (3) 0 V → −2 V, and (4) −2 V → 0 V. In each sample, the *I*_S_-*V*_S_ curves were acquired 15–20 times on each NC and measured NC number was 6–8 for each sample (total *I*_S_-*V*_S_ curves: 120 for each sample). Figure 2(c) is the *I*_S_-*V*_S_ curve of as-grown NCs with Fe coating layer of 30 MLs. The NCs showed the bipolar-type resistive switching characteristics with high resistive state (HRS) and low resistive state (LRS). The resistive switching probability was estimated by defining as the switching observation number divided by measurement number (Supplemental Material 2). The average resistive switching probabilities in one sample (*P*_switch_) were ~18, ~32, and ~43% in Fe_3_O_4_ NCs on Ge nuclei with Fe coating layers of 6, 15, and 30 MLs, respectively (Figure 2(d)). The Fe coating amount dependence of the crystallinity and the *P*_switch_ demonstrated that *P*_switch_ becomes higher as the crystallinity of Fe_3_O_4_ NCs is enhanced. In NCs with lower crystallinity, it can be considered that there are more defects (oxygen vacancies) in Fe_3_O_4_. The carrier concentration can be increased in Fe_3_O_4_ due to more oxygen vacancies, enhancing the screening effect of applied electric field in Fe_3_O_4_. As a result, the electric-field-induced oxygen vacancy movement becomes difficult to occur in Fe_3_O_4_, resulting in low *P*_switch_ in defective samples (Figure 2(e)). On the other hand, in NCs with higher crystallinity, the electric-field-induced oxygen vacancy movement likely occurs due to the small screening effect, resulting in higher *P*_switch_ (Figure 2(e)).

### 3.2. Composition and structural analyses of annealed NCs

**Figure 3.**
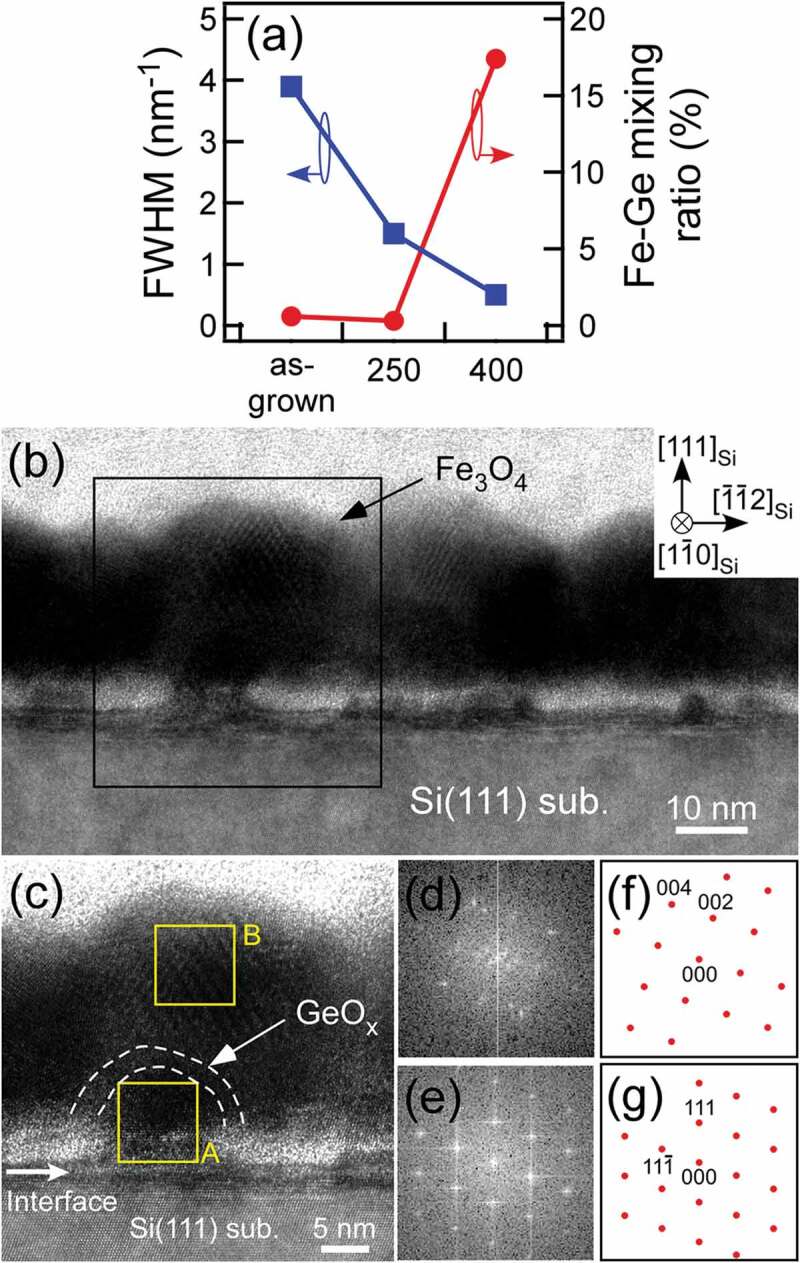
(a) FWHM estimated from 224_Fe3O4_ peak in RHEED (left axis) and Fe-Ge mixing ratio measured from XPS spectra (right axis) in each sample (as-grown: as-grown NCs, 250: NCs annealed at 250°C, and 400: NCs annealed at 400°C). (b) Low-magnification HRTEM image of NCs annealed at 250°C. (c) Enlarged HRTEM image of the square region in (b). FFT patterns of (d) regions B and (e) A in (c). The region marked by the broken line denotes GeO_x_. Theoretical FFT patterns of (f) Fe_3_O_4_ and (g) Ge

To enhance the crystallinity of Fe_3_O_4_ NCs, as-grown NCs were post-annealed under an oxygen pressure of 2 × 10^−4^ Pa, where the two post-annealing temperatures were used: 250°C and 400°C. The analyses of 224_Fe3O4_ spots in RHEED patterns were performed by fitting with Gaussian function. The FWHM values of the 224_Fe3O4_ fitted peaks in the NCs annealed at 250°C and 400°C were 1.5 and 0.5 nm^−1^, respectively, indicating that the crystallinity of Fe_3_O_4_ was enhanced as post-annealing temperature became higher (Figure 3(a)). The compositions of as-grown NCs and the NCs annealed at 250°C and 400°C were investigated by XPS measurements in the Fe2p region (Supplemental Material 3). Other studies have reported that FeO and *γ*-Fe_2_O_3_ have satellite peaks near 715 or 720 eV, respectively, while Fe_3_O_4_ has no satellite peaks [30]. The XPS spectra of as-grown NCs and the NCs annealed at 250°C and 400°C displayed no satellite peaks, indicating that all the samples are Fe_3_O_4_. We also measured the XPS spectra in the Ge3d region to obtain the compositional information near the Fe_3_O_4_/Ge interface (Supplemental Material 3). All the NCs had the peaks coming from oxidation states of Ge, proving that GeO_x_ was formed at the interface between Fe_3_O_4_ and Ge nuclei. In the sample post-annealed at higher temperature (NCs annealed at 400°C), remarkable Fe-Ge peaks were also observed. The XPS spectra in the Ge3d region were deconvolved with Gaussian function at the peak positions of Ge^0^ (29.3 eV), Ge^+^ (30.1 eV), Ge^2+^ (31.1 eV), Ge^3+^ (32.0 eV), Ge^4+^ (32.6 eV), and Fe-Ge (29.8 eV) [31,32]. The integrated intensity ratios of Fe-Ge peak to the sum of all the peaks (Fe-Ge mixing ratio) were almost zero for as-grown (~0.6%) and NCs annealed at 250°C (~0.3%), while the ratio was high (17.4%) for NCs annealed at 400°C (Figure 3(a)). In NCs annealed at 400°C, this high Fe-Ge mixing ratio implies that Fe-Ge mixing occurred at the region near the interface, namely in GeO_x_ interface layer, during high-temperature annealing process. This demonstrates that as-grown NCs and NCs annealed at 250°C without high-temperature annealing process have higher-quality GeO_x_ with almost no Fe-Ge mixing than NCs annealed at 400°C.

Figure 3(b) shows a cross-sectional HRTEM image of NCs annealed at 250°C with high-crystallinity Fe_3_O_4_ and high-quality GeO_x_. It was confirmed that NCs with small size of ~30 nm were formed, which is consistent with the result of AFM observation. Figure 3(c) is an enlarged image of the square region in Figure 3(b). The crystal structures in the regions A and B in Figure 3(b) were analysed by fast Fourier transformation (FFT). As shown in Figure 3(d) and (e), the FFT patterns of regions B and A were consistent with the theoretical diffraction ones of Fe_3_O_4_ and Ge with the epitaxial relationship of (012)_Fe3O4_//(111)_Ge_ (Figure 3(f) and (g)), respectively, demonstrating that Fe_3_O_4_ shell/Ge core NCs were epitaxially formed. In addition, the bright contrast region was observed between Fe_3_O_4_ shell and Ge core, as indicated by the arrow in the HRTEM image (Figure 3(c)), implying that amorphous GeO_x_ exists. Thus, HRTEM observations revealed the structures of NCs: Fe_3_O_4_/GeO_x_/Ge NCs.

### 3.3. Resistive switching characteristics in annealed NCs

**Figure 4.**
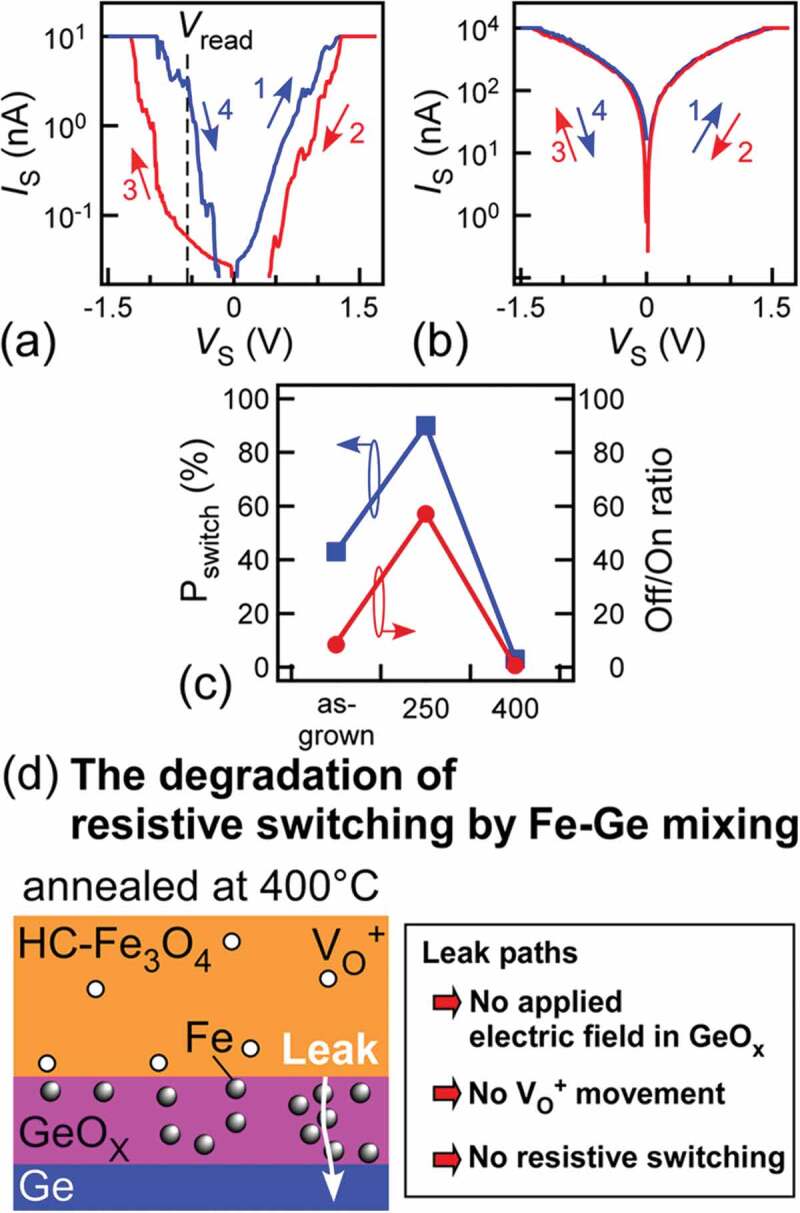
The *I*_S_-*V*_S_ curves of (a) NCs annealed at 250°C and (b) at 400°C. (c) *P*_switch_ and Off/On ratio of all the NCs. The solid squares and circles denote the *P*_switch_ and the Off/On ratio, respectively. (d) Schematic of the GeO_x_ quality effect on the resistive switching characteristics

In C-AFM measurements, bipolar-type resistive switching characteristics were observed in as-grown NCs (Figure 2(c)) and NCs annealed at 250°C (Figure 4(a)), while the resistive switching seldom occurred in NCs annealed at 400°C (Figure 4(b)). By considering there is Fe-Ge mixing (low-quality GeO_x_) only in NCs annealed at 400°C, it was found that the unobserved resistive switching characteristics in NCs annealed at 400°C were attributed to the existence of FeGe alloys in GeO_x_ although NCs annealed at 400°C have higher-crystallinity Fe_3_O_4_ than other NCs. To quantitatively investigate the nanostructure interface effect on the resistive switching characteristics, we measured *P*_switch_ and Off/On ratio, where the *I*_S_-*V*_S_ curves were acquired 15–20 times on each NC and measured NC number was 6–8 for each sample (total *I*_S_-*V*_S_ curves: 120 for each sample). The *P*_switch_ values were ~43, ~90, and ~3% in as-grown NCs, NCs annealed at 250°C and 400°C, respectively (Figure 4(c) and Supplemental Material 2). Note that NCs annealed at 250°C exhibited substantially higher *P*_switch_ (~90%) than those of the other NCs. Besides, the standard deviation of the resistive switching probabilities of individual NCs in the NC sample annealed at 250°C (Supplemental Material 2) was 0.09, which was extremely smaller than those of as-grown NCs (0.26) and previous Fe_3_O_4_ NCs formed at high temperature (0.3). This indicates that the resistive switching uniformity was improved by forming high-crystallinity Fe_3_O_4_ and high-quality GeO_x_. The NCs annealed at 250°C exhibited higher Off/On ratio (~58) at a reading *V*_S_ (*V*_read_) of ~ −0.5 V than those of the other NCs: ~0 for NCs annealed at 400°C and ~9 for as-grown NCs (Figure 4(c)). During the *I*_S_-*V*_S_ measurement cycles, the HRS resistance gradually got larger, indicating that the NCs memorize the current history like memristor. From its higher *P*_switch_ and Off/On ratio, the NCs annealed at 250°C are most suited for NC memory among them. Compared with as-grown NCs, high *P*_switch_ and Off/On ratio in NCs annealed at 250°C can be attributed to the low concentration of oxygen vacancies; this relates to the higher Fe_3_O_4_ crystallinity, which can be explained by the same mechanism as for as-grown NCs (Figure 2(e)). Another remarkable fact is that the formation of FeGe alloys in GeO_x_ prevented the resistive switching (Figure 4(d)). In the NCs with low-quality GeO_x_ (including FeGe alloys), the electric field is not applied to GeO_x_ because FeGe alloys in GeO_x_ work as current leak paths. It should be noted that the resistive switching characteristics were affected by character of Fe_3_O_4_ and GeO_x_, implying that HRL related to resistive switching is composed of both GeO_x_ and Fe_3_O_4_ near Fe_3_O_4_/GeO_x_ interface. For further enhancement of resistive switching performance, almost perfect stoichiometric Fe_3_O_4_ and high-quality GeO_x_ without current leak paths are key structures.

The resistive switching mechanism in this system can be discussed below. In the bipolar-type resistive switching, there are mainly two mechanisms such as filament- or interface-type. If the resistive switching mechanism in the Fe_3_O_4_ is filament-type, the resistive switching can be caused in Fe_3_O_4_ films without Ge. However, in our previous study, resistive switching was not observed in conventional pulse laser deposition Fe_3_O_4_ films on Si substrate without Ge [17]. The quality of the pulse laser deposition films, which is related to some defects (oxygen vacancy concentration, point defect species), is similar to that of the present Fe_3_O_4_/Ge NCs because of the same growth condition of low oxygen pressure (10^−4^ Pa). Therefore, the filament-type mechanism is ruled out in the present system. On the other hand, in terms of the deficiency, the quality of pulse laser deposition films and the present Fe_3_O_4_/Ge NCs can be quite different from the reported films formed by sputtering method exhibiting resistive switching [19,20].

In the case of interface-type resistive switching, there are several candidates of resistive switching interface: Pt-Ir cantilever/Fe_3_O_4_, Fe_3_O_4_/GeO_x_, and GeO_x_/Ge. The C-AFM observations in air might cause the anodic oxidation of NC surface under Pt-Ir cantilever, bringing the resistance change when applying negative *V*_S_. In the same mechanism, Fe_3_O_4_ films without Ge must show resistive switching behavior. In our previous study, the pulse laser deposition Fe_3_O_4_ films without Ge did not occur. Therefore, the resistive switching by anodic oxidation is ruled out. Next, we discuss whether the resistive switching at the GeO_x_/Ge interface can be caused or not. In our previous study [25], Ge nuclei covered with GeO_x_, namely the sample without Fe_3_O_4_, did not exhibit resistive switching, indicating that the GeO_x_/Ge is not a resistive switching interface in the present system. Therefore, the possible resistive switching interface is narrowed down to the Fe_3_O_4_/GeO_x_.

We consider the HRL, where the electric field is mainly applied when applying *V*_S_ to NCs. For resistive switching, the electric field application to GeO_x_ is required because the resistive switching did not occur when the electric field cannot be applied to GeO_x_ due to the formation of FeGe alloys working as current leak paths. This indicates that GeO_x_ is a part of HRL. In addition, the resistance of Fe_3_O_4_ should be relatively high because the resistive switching performance becomes higher with enhancing Fe_3_O_4_ crystallinity. This indicates that the electric field should also be applied to Fe_3_O_4_ near Fe_3_O_4_/GeO_x_ interface. From these results, it is considered that HRL is composed of both GeO_x_ and Fe_3_O_4_ near Fe_3_O_4_/GeO_x_ interface. However, we believe that the main contribution of the HRL resistance comes from GeO_x_ because resistive switching seldom occurred at zero electric field in GeO_x_ in NCs annealed at 400°C.

**Figure 5.**
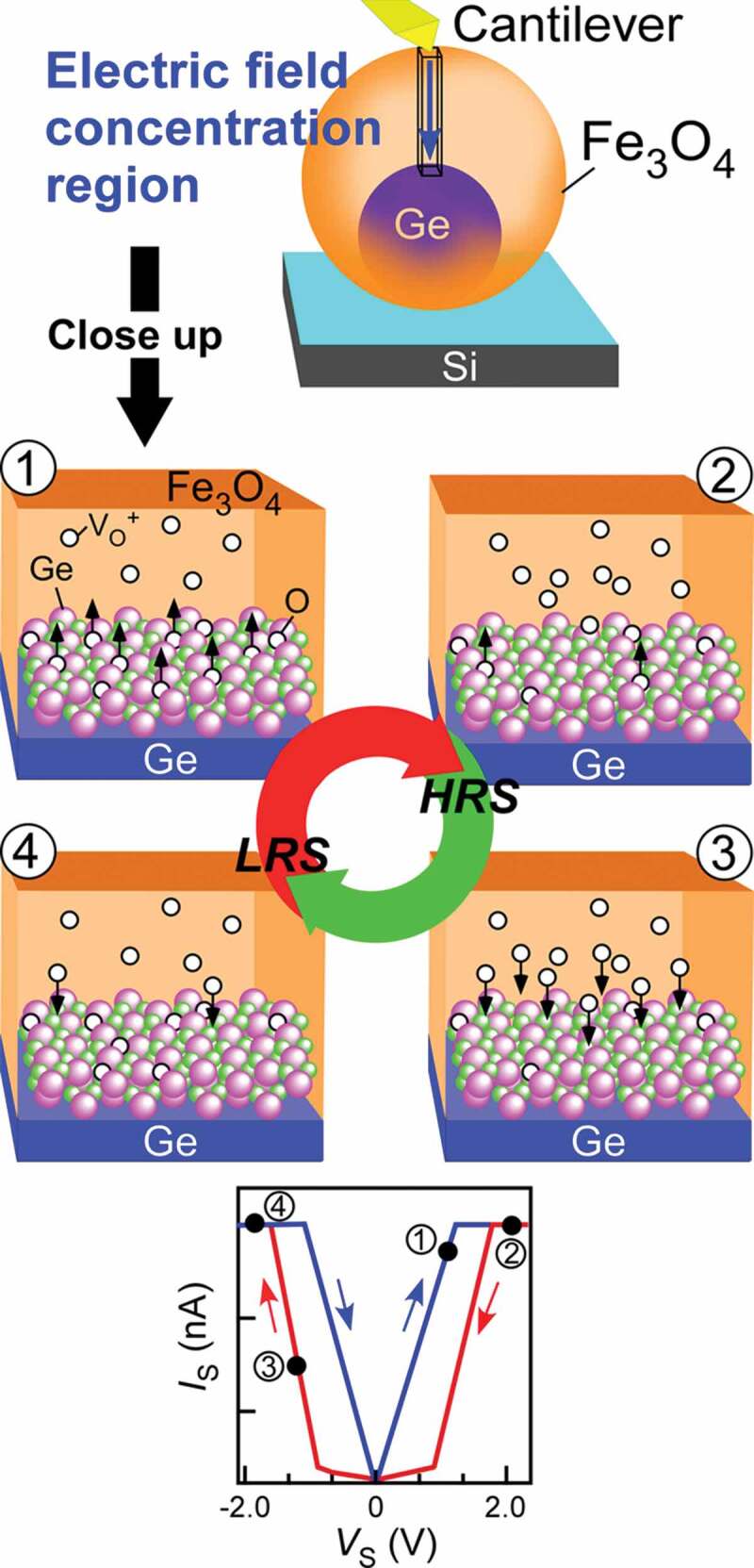
Resistive switching mechanism in Fe_3_O_4_/GeO_x_/Ge NCs

As shown in Figure 5, when applying *V*_S_ to NCs, positively charged oxygen vacancies move in GeO_x_ and Fe_3_O_4_ near Fe_3_O_4_/GeO_x_ interface. When the x value of GeO_x_ gets larger (smaller) by this ionic movement, the volume of the high resistance GeO_x_ with large x becomes larger (smaller) [33] and the HRL thickness composed of high resistance GeO_x_ increases (decreases). On the other hand, as the effect of the electric field applied to Fe_3_O_4_ near Fe_3_O_4_/GeO_x_ interface, two possibilities are considered: (1) changing resistance states of Fe oxide layer near Fe_3_O_4_/GeO_x_ interface: LRS of Fe_3_O_4_ and HRS such as *γ*-Fe_2_O_3_ or FeGeO_x_ by the ionic movement, (2) supplying oxygen vacancies or oxygens from Fe_3_O_4_ region to GeO_x_. By either function, the electric-field resistive change is enhanced. This deep comprehension about the resistive switching mechanism is presenting the material design guideline for improving various resistive switching characteristics.

### 3.4. Direct observation of memory effect

**Figure 6.**
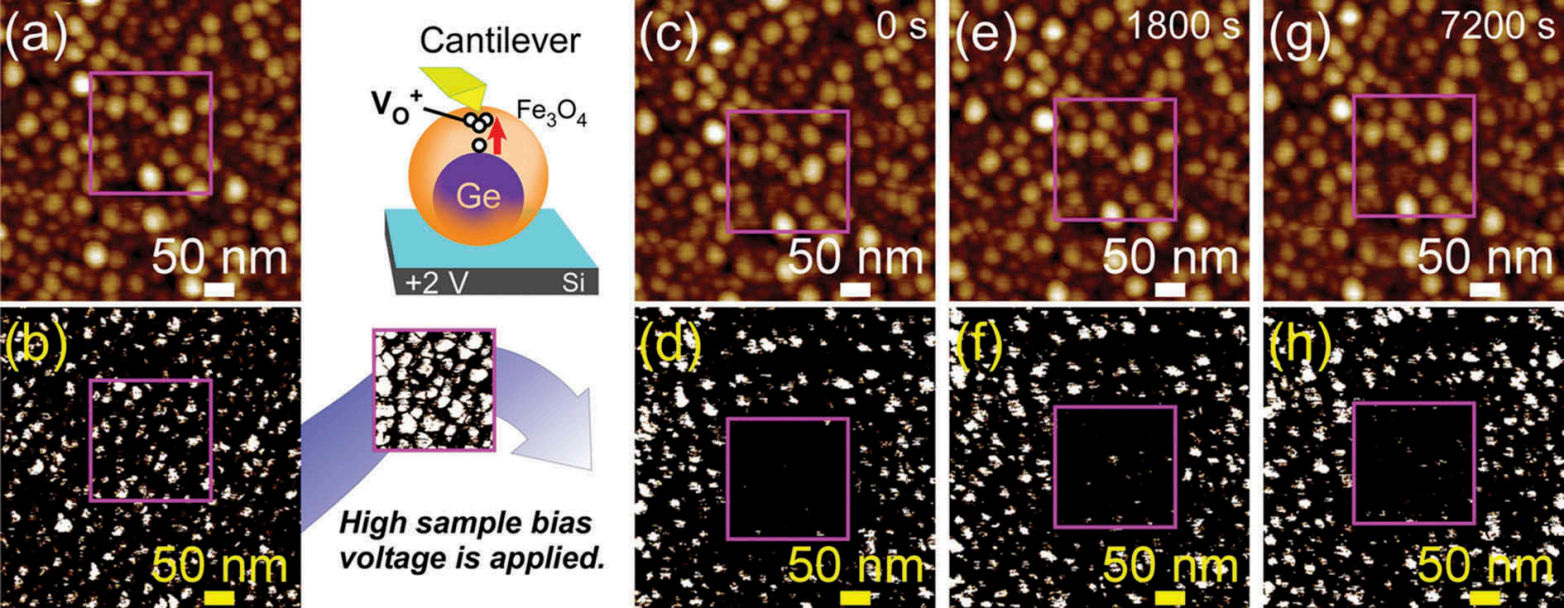
Memory effect of NCs annealed at 250°C (a, c, e, g) AFM and (b, d, f, h) current mapping images of the NCs at the *V*_S_ of −0.5V: (a, b) before applying *V*_S_ of +2V, (c, d) just after applying *V*_S_ of +2V, (e, f) 1800 s later since the resistive switching, and (g, h) 7200 s later since the resistive switching, respectively. The current mapping image when applying *V*_S_ of +2V is shown in the inset

Here, the memory effect in NCs annealed at 250°C was directly observed by performing current mapping with C-AFM. Figure 6(a) and (b) show AFM and current mapping images, respectively, before applying the *V*_S_ of +2 V. The current mapping images were observed at the *V*_read_ of −0.5 V, which applied voltage does not cause the resistive switching. White contrasts indicate the higher electric currents between cantilever and sample. Almost all NCs show white contrasts in current mapping images, which indicates that the initial states of NCs were LRS. We applied voltages to the samples by scanning the square region of Figure 6(b) at *V*_S_ of +2 V to cause the resistive switching. After applying the *V*_S_, the white contrasts in the square region disappeared and most of the NC sites inside the square region became darker compared with other NCs outside the region (Figure 6(c) and (d)). Thus, it was directly observed that the resistance of NCs in the square region changed from LRS to HRS through the application of *V*_S_ of +2 V. AFM and a current mapping image were observed at the *V*_read_ of −0.5 V (Figure 6(e) and (f)) when the time of 1800 s passed after the resistive switching by scanning at *V*_S_ of +2 V. The results demonstrate that the contrast in square region was kept darker, indicating that the resistance in NCs has been memorized since the resistive switching (LRS to HRS). We also confirmed the resistance retention of more than 7200 s in such C-AFM method (Figure 6(g) and (h)). The direct observation of the memory effect in NCs demonstrates that NCs annealed at 250°C have a high potentiality as a non-volatile memory. Unlike the film memory material, the observation of the memory effect in ultrasmall NCs has never been reported in ReRAM. The experimental confirmation of the memory effect existence even in ultrasmall NCs is significant for realizing non-volatile NC memory leading to neuromorphic device.

## 4. Conclusions

We developed high-density and isolated Fe_3_O_4_/GeO_x_/Ge NCs with high-performance resistive switching characteristics by low-temperature growth. Therein, the quality of the interfaces; the crystallinity of Fe_3_O_4_ and the quality of GeO_x_ were successfully enhanced by annealing under proper condition: low oxygen pressure (2 × 10^−4^ Pa). The high quality of interfaces enables effective application of electric field to Fe_3_O_4_ and GeO_x_. Then, the electric field induces positively charged oxygen vacancy movement, leading to the oxidation degree change of HRL composed of Fe oxide and GeO_x_. As a result, the thickness of HRL can be changed by applying the electric field effectively. The NCs annealed at 250°C showed the bipolar resistive switching characteristics with higher *P*_switch_ of ~90% and higher Off/On ratio of ~58. The memory effect in NCs annealed at 250°C was also observed by C-AFM method successfully. The experimental confirmation of the memory effect existence even in ultrasmall NCs is significant for realizing non-volatile NC memory leading to neuromorphic device.

## Supplementary Material

Supplemental Material
