# Supplementary material for: Resistive switching memory performance in oxide hetero-nanocrystals with well-controlled interfaces
Source: Sci Technol Adv Mater. 2020 Mar 19;21(1):195–204. doi: 10.1080/14686996.2020.1736948 (PMC7144302; doi:10.1080/14686996.2020.1736948)
Supplement: Supplemental Material [file TSTA_A_1736948_SM2915.pdf]

Supplemental Material: Resistive switching memory performance in oxide hetero-nanocrystals with well-controlled interfaces

Takafumi Ishibe[a], Yoshiki Maeda[a], Tsukasa Terada[a], Nobuyasu Naruse[b], Yutaka Mera[b], Eiichi Kobayashi[c], and Yoshiaki Nakamura[a]

[a]*Graduate School of Engineering Science, Osaka University, 1-3 Machikaneyama-cho, Toyonaka, Osaka 560-8531, Japan*

[b]*Department of Fundamental Bioscience, Shiga University of Medical Science, Otsu, Shiga 520-2192, Japan*

[c]*Kyushu Synchrotron Light Research Center, 8-7 Yayoigaoka, Tosu, Saga 841-0005, Japan*

E-mail: nakamura@ee.es.osaka-u.ac.jp

Section S1: The relationship between Fe_3O_4 crystallinity and Fe deposition amount

To compare the crystallinities of Fe_3O_4/GeO_x/Ge nanocrystals (NCs) with Fe coating layers of 6, 15, and 30 MLs, the analyses of 224_{Fe3O4} spots in reflection high energy electron diffraction (RHEED) patterns of these NCs (Figs. S1(a)-(c)) were performed by fitting with Gaussian function. Figures S1(d)-(f) show the line profiles corresponding to the solid lines in Figs. S1(a)-(c), respectively. The full width at half maximum (FWHM) values of the 224_{Fe3O4} fitted peaks in Fe_3O_4/Ge NCs with Fe coating layers of 6, 15, and 30 MLs were acquired to be 5.5, 4.7, and 3.9 nm^{-1}, respectively, indicating that the crystallinity is enhanced with increasing the deposition amount of Fe coating layer. This relationship between Fe_3O_4 crystallinity and Fe deposition amount is similar to the previously-reported result in Fe_3O_4 NCs on Ge nuclei formed by Fe deposition at high temperature of 250ºC [1], where it was reported that oxidation of Ge nucleus surface was prevented at the first stage of Fe_3O_4 growth by Fe coating, resulting in the higher crystallinity of Fe_3O_4.

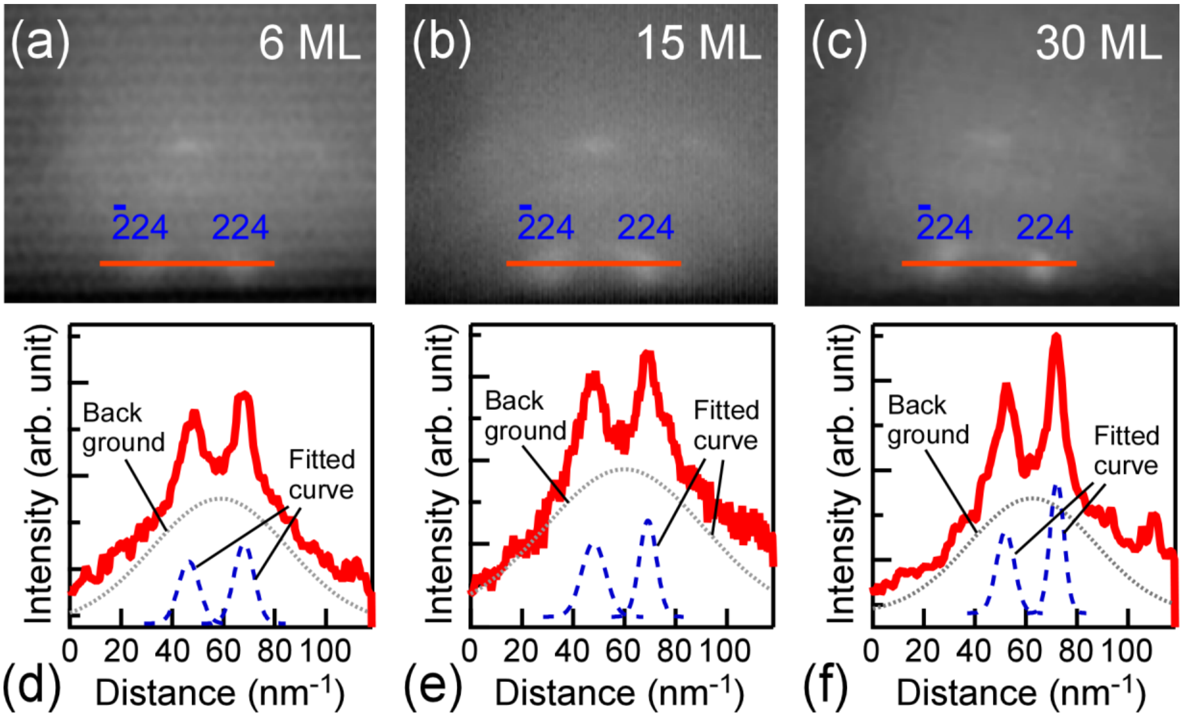

Figure S1 The RHEED patterns of Fe_3O_4 NCs on Ge nuclei with Fe coating layers of (a) 6, (b) 15, and (c) 30 MLs, respectively. The profiles along the lines in (a), (b) and (c) are shown in (d), (e), and (f) respectively. The dotted and broken curves denote background and fitted ones, respectively.

Section S2: The resistive switching variability of the NCs

The I_S-V_S curves were acquired 15-20 times on each NC by C-AFM and measured NC number was 6-8 for each sample (total I_S-V_S curves: 120 for each sample). The resistive switching probability value was defined as the switching observation number divided by measurement number. The resistive switching probability value measured on each NC is shown in Table S1. The average resistive switching probability value in one sample (P_{switch}) was ~43, ~90, and ~3 % in as-grown NCs and NCs annealed at 250ºC and at 400ºC, respectively (Fig. S2). Note that NCs annealed at 250ºC exhibited substantially higher P_{switch} of ~90 % than the other NCs. Besides, the standard deviations of the P_{switch} of individual NCs were estimated from Table S1 to be 0.09 in NCs annealed at 250ºC, 0.26 in as-grown NCs. For reference, that in our previous Fe_3O_4 NCs formed at high temperature is 0.3. This demonstrates that NCs annealed at 250ºC had that the resistive switching uniformity was improved.

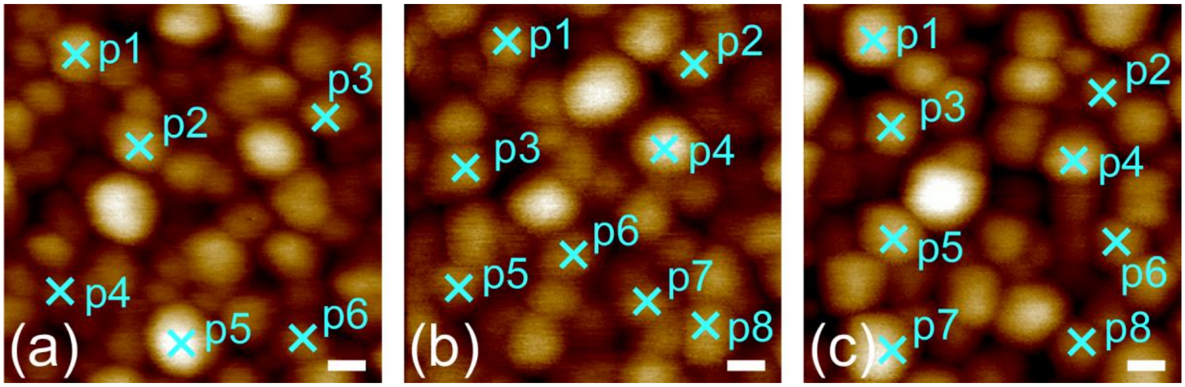

Figure S2 AFM images of (a) as-grown NCs and (b) NCs annealed at 250ºC and (c) at 400ºC. The I_S-V_S measurement points are denoted by the cross marks in (a)-(c). Solid lines denote scale bars of 20 nm.

Table S1 Resistive switching probability measured on each NC in each sample.

	p1	p2	p3	p4	p5	p6	p7	p8
as-grown	45%	30%	45%	5%	50%	85%		
250	100%	87%	87%	93%	73%	87%	93%	100%
400	7%	0%	7%	0%	0%	7%	0%	0%

Section S3: XPS measurement of Fe_3O_4 NCs with the interfaces of $Fe_3O_4/GeO_x/Ge$

The compositions of as-grown NCs and NCs annealed at 250ºC and 400ºC were precisely investigated by XPS measurements in the Fe2p region (Fig. S3). Other studies have reported that FeO and γ-Fe_2O_3 have satellite peaks near 715 or 720 eV, respectively, while Fe_3O_4 has no satellite peaks [2]. The XPS spectra of as-grown NCs and NCs annealed at 250ºC and 400ºC displayed no satellite peaks, indicating that all the samples are Fe_3O_4. We also measured the XPS spectra around Ge3d to obtain the compositional information near the interface (Fig. S4). All the NCs had the peaks coming from oxidation states of Ge, indicating that GeO_x was formed between Fe_3O_4 and Ge nuclei through the oxidation of Ge nucleus interface. In the sample annealed at higher temperature (NCs annealed at 400ºC), remarkable Fe-Ge peaks were also observed. The XPS spectra in the Ge3d region were deconvolved with Gaussian functions at the peak positions of Ge^0 (29.3 eV), Ge^+ (30.1 eV), Ge^{2+} (31.1 eV), Ge^{3+} (32.0 eV), Ge^{4+} (32.6 eV), and Fe-Ge (29.8 eV) [3,4]. The integrated intensity ratios of Fe-Ge peak to the sum of all the peaks (Fe-Ge mixing ratio) were almost zero for as-grown (~0.6%) and NCs annealed at 250ºC (~0.3%), while the ratio was high (17.4%) for NCs annealed at 400ºC (Fig. 3(a) in main text). In NCs annealed at 400ºC, this high Fe-Ge mixing ratio implies that Fe and Ge were mixed at the region near the interface, namely in GeO_x interface layer, during high temperature annealing process. This demonstrates that as-grown NCs and NCs annealed at 250ºC without high temperature annealing process have higher-quality GeO_x than NCs annealed at 400ºC.

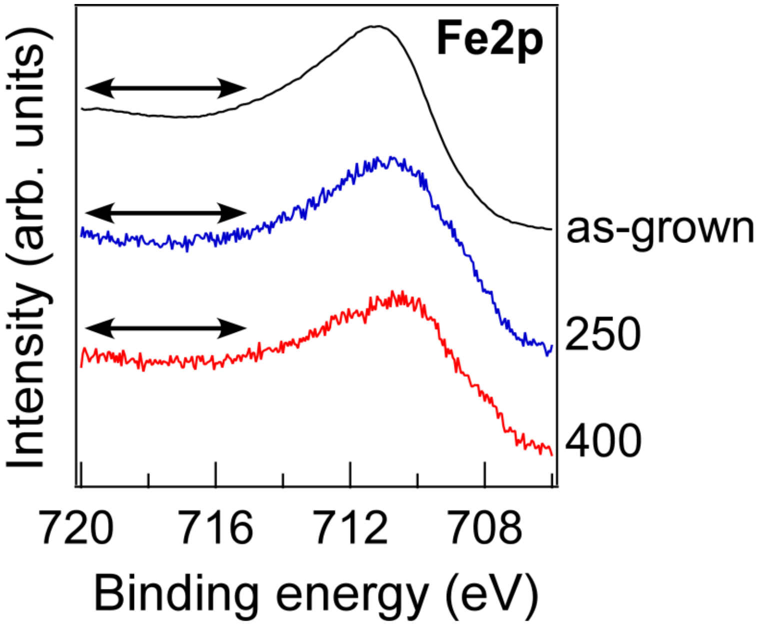

Figure S3 XPS spectra in the Fe2p region in as-grown NCs and NCs annealed at 250ºC (250) and NCs annealed at 400ºC (400). No satellite peaks near 715-720 eV.

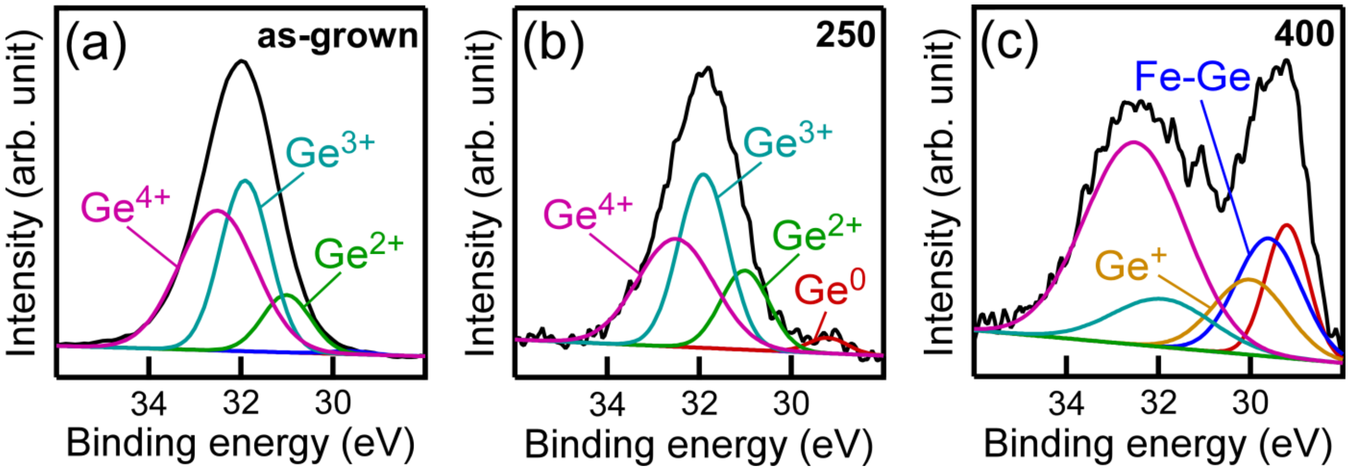

Figure S4 XPS spectra around Ge3d in (a) as-grown NCs and (b) NCs annealed at 250ºC (250) and (c) at 400ºC (400).

References

[1] Ishibe T., Watanabe K., Nakamura Y. Effect of Fe coating of nucleation sites on epitaxial growth of Fe oxide nanocrystals on Si substrates. Jpn J Appl Phys. **2016**;55:08NB12.

[2] Venkateshvaran D., Kaiser W., Boger A., et al. Anomalous Hall effect in magnetite: Universal scaling relation between Hall and longitudinal conductivity in low-conductivity ferromagnets. Phys Rev B **2008**;78,092405.

[3] Ruby C., Humbert B., Fusy J. Surface and interface properties of epitaxial iron oxide thin films deposited on MgO(001) studied by XPS and Raman spectroscopy. Surf Interface Anal. **2000**;29:377-380.

[4] Venugopal R., Sundaravel B., Wilson I. H., et al. Structural and magnetic properties of Fe-Ge layer produced by Fe ion-implantation into germanium. J Appl Phys. **2002**;91:1410-1416.
